# Neurocognition and Social Cognition— Possibilities for Diagnosis and Treatment in Ultra-High Risk for Psychosis State

**DOI:** 10.3389/fpsyt.2021.765126

**Published:** 2021-11-22

**Authors:** Katarzyna Rek-Owodziń, Ernest Tyburski, Katarzyna Waszczuk, Jerzy Samochowiec, Monika Mak

**Affiliations:** ^1^Department of Health Psychology, Pomeranian Medical University in Szczecin, Szczecin, Poland; ^2^Department of Psychiatry, Pomeranian Medical University in Szczecin, Szczecin, Poland

**Keywords:** neurocognition, social cognition, ultra-high risk for psychosis, schizophrenia prodrome, schizophrenia spectrum, diagnosis, treatment

## Abstract

In recent decades, clinicians have developed the construct of ultra-high risk (UHR) for psychosis to characterize the prodromal phase of psychosis or classify people with weakly expressed psychotic symptoms. In this conceptual analysis, we have gathered up-to-date data about the clinical picture of neurocognition and social cognition in people at UHR for psychosis. We also discuss treatment options. A well-chosen therapeutic approach can help to deal with difficulties and delay or even prevent the development of full-blown psychotic disorders in the UHR group. Despite much evidence supporting the benefits of therapy, early interventions are still not as widely used as they should be. Thus, a better understanding of the UHR state is very important for all healthcare workers.

## Introduction

In recent decades, clinicians have developed the construct of ultra-high risk (UHR) for psychosis states. This condition is associated with the risk of a diagnosis of schizophrenia or other psychotic disorder within 1-3 years of manifestation of symptoms and is sometimes considered the prodromal phase of psychosis (1). Some people at UHR will not develop a full-blown illness, but their symptoms can cause distress and affect every day functioning (2, 3).

Specific deficits in cognitive functioning in schizophrenia were reported a long time ago by the pioneers Kraepelin (4) and Bleuler (5). Furthermore, they are recognized as core features of this severe psychotic disorder (6). A number of studies have shown that subjects in the prodromal phase of psychosis show overall impairments in cognitive functioning, but these are substantially less severe than in first-episode psychosis (FP) and chronic schizophrenia (CHS) (6, 7). Some findings even suggest that cognitive deficits may already exist before the development of symptoms of UHR (8). However, it is not clear whether deterioration of cognitive functions always co-occurs with the increasing severity of psychotic symptoms during the transition from UHR to FP (8). Neurocognitive deficits are not only characteristic of the UHR condition—studies indicate that patients in the prodromal phase of psychosis present also deficits in social cognition (9). Specific dysfunctions in neuro- and social cognition lead to difficulties in various areas of life, such as education, work, social life, mood.

Detailed study of the UHR state is important not only from the perspective of clinical knowledge, but also for the planning of interventions for people with early psychotic states. Fast diagnosis and well-chosen therapy can reduce suffering and slow down or even prevent the development of full-blown psychosis or other disorders in the UHR group. This group of patients requires an extremely delicate approach because of the risk of stigmatization and its negative consequences. Knowledge about different aspects of UHR for psychosis should be widespread among healthcare workers, as this would improve outcomes for people affected by this condition.

Therefore, this article aims to present the primary characteristics of the UHR state, the associated neurocognition and social cognition, and possibilities for treatment, taking into consideration the controversy in this field. There is a need to set an unambiguous direction in thinking about people with the UHR state.

## Clinical Characteristics of UHR for Psychosis

Psychotic disorders are not binary conditions, but rather they occur on a spectrum. In the 19th and 20th centuries, clinicians and researchers focused on establishing proper criteria and treatment recommendations for people with severe and long-lasting psychotic symptoms. Detailed analysis of the medical history of many patients showed that specific prodromal symptoms occurred before the development of a full picture of the disease. Furthermore, young people with concurrent psychotic symptoms who did not fit in any particular unit in an institution were increasingly referred to specialists. Following the need to fully understand different stages of psychotic spectrum disorders, clinicians developed the construct of UHR for psychosis. Other definitions of UHR exist, though with some theoretical differences (10).

For over 20 years, the criteria for UHR for psychosis have been used worldwide due to their predictive validity (11). However, this state is not described in any leading classifications such as ICD-10 or DSM V. It was used by clinical practitioners and researchers to catch people in very early phases of the psychotic spectrum. To meet the criteria of UHR, one or more of the following must be present: attenuated psychotic symptoms (APS), a brief limited intermittent psychotic episode (BLIPS), trait vulnerability plus a marked decline in psychosocial functioning, known as genetic risk and deterioration syndrome (GRD), or unspecified prodromal symptoms (UPS) (10). APS must be present at least several times a week within the past year (but no longer than 5 years) and must include psychotic symptoms of sufficient severity and frequency as ideas of reference, perceptual disturbances, odd beliefs or magical thinking, paranoid ideation, odd thinking and speech, and odd behavior and appearance (12, 13). BLIPS—understood as frank, transient psychotic symptoms—must be present for at least several minutes a day, but cannot last longer than a week, at a frequency of at least once per month, and cannot be described by another disorder (12). BLIPS symptoms resolve spontaneously (13). GRD can be diagnosed when a person meets the criteria for a schizotypal personality disorder or when one has a first-degree relative with a psychotic disorder and significant decline in mental state or functioning have been present for at least 1 month (13).

Prodromal symptoms may also include neurocognitive dysfunctions, but their presence is not necessary for the diagnosis. UHR criteria may be measured by different scales, most of which are based on detailed interviews, as for example: the Structured Interview for Prodromal Symptoms (SIPS), the Comprehensive Assessment of At-Risk Mental States (CAARMS), the Basel Screening Instrument for Psychosis (BSIP), and short screening methods such as the Prodromal Questionnaire - Brief version (PQ-B) or Prime Screen—Revised (PRIME) (10, 12, 14).

Several studies have been conducted to estimate the risk of transition to psychosis in the UHR group. Different results have been obtained. Fusar-Poli et al. (1) in their meta-analysis, showed that individuals with a UHR diagnosis have a 15-30% risk of developing a psychotic disorder within 12 months and after 3 years this grows to over 36%. Earlier studies tended to show higher rates of transition than later studies. This may be an instance of the decline effect - the phenomenon of fewer new studies supporting a hypothesis as time progresses (15).

Despite meeting the criteria for UHR, an individual may never meet the criteria of any psychotic disorder or other psychiatric diagnosis. It is very important to be extra careful making a diagnosis to avoid stigmatization and iatrogenic effects in such a sensitive population. A recent study indicates that 43% of patients in the UHR sample recover or go into remission from UHR and 57% have no remission, transition, or relapse over 1 year (16). Some help-seekers diagnosed with UHR ultimately get a diagnosis of another (not psychotic) disorder, such as depression, bipolar disorder, personality disorder, or substance abuse (16). On the other hand, after diagnosis of the prodromal phase of psychosis, frequent evaluation of symptoms is needed to catch a potential transition to psychosis.

The criteria for transition are not clearly defined. Some of authors define them as the occurrence of at least 1 fully positive psychotic symptom which is present at least several times a week for more than 1 week or the presence of at least 1 full psychotic symptom for at least 1 day if this symptom is seriously disorganizing or dangerous (10, 16, 17). The differences between UHR, schizophrenia, acute psychotic disorder, and other psychotic disorders mostly concern the severity, duration, and frequency of psychotic symptoms (Table 1). In some cases, mental states can change rapidly, otherwise the process of transition may last years and be gradual.

**Table 1:** Diagnostic criteria of ultra-high risk (12), schizophrenia (18) and acute transient disorder (18).

	**Ultra-high risk**	**Schizophrenia**	**Acute and transient psychotic disorders**
Symptoms	- At least one of the following: ideas of reference, magical thinking, perceptual disturbance, paranoid ideation, odd thinking or speech, or Trait and state risk factors and significant decline in mental state or functioning	- At least one of the following: (a) Thought echo, thought insertion or withdrawal, or thought broadcasting; (b) Delusions of control, influence or passivity, clearly referred to body or limb movements or specific thoughts, actions, or sensations; delusional perception; (c) Hallucinatory voices giving a running commentary on the patient's behavior, or discussing him between themselves, or other types of hallucinatory voices coming from some part of the body; (d) Persistent delusions of other kinds that are culturally inappropriate and completely impossible (e.g. being able to control the weather, or being in communication with aliens from another world), or - At least two of the following: (e) Persistent hallucinations in any modality, when occurring every day for at least one month, when accompanied by delusions (which may be fleeting or half-formed) without clear affective content, or when accompanied by persistent over-valued ideas; (f) Neologisms, breaks or interpolations in the train of thought, resulting in incoherence or irrelevant speech; (g) Catatonic behavior, such as excitement, posturing or waxy flexibility, negativism, mutism and stupor; (h) “Negative” symptoms such as marked apathy, paucity of speech, and blunting or incongruity of emotional responses (it must be clear that these are not due to depression or to neuroleptic medication)	- Delusions, hallucinations, and perceptual disturbances, and severe disruption of ordinary behavior
Duration of symptoms	- At least 1 week and no longer than 5 years for APS - <1 week for BLIPS - At least 1 month and not longer than 5 years for GRD	- At least 1 month	- Acute onset, 2 weeks or less
Frequency of symptoms	- At least several times a week	- Most of the time during 1 month	- Most of the time during episode

*APS, attenuated psychotic symptoms. BLIPS, brief limited intermittent psychotic episode. GD, genetic risk and deterioration syndrome*.

The precise prevalence of UHR for psychosis in populations is unknown because the diagnostic criteria were established based on research conducted on samples of people who had sought help (10). Data obtained in various studies estimates the prevalence of psychotic symptoms or psychotic-like experiences to be around 4-8%—the results varied depending on the method of diagnosis (structured interviews, questionnaires, or clinical interviews) (10, 19). Children and adolescents may report psychotic symptoms which do not cause significant distress and go into remission without any medical interventions (for example: suspiciousness, bizarre behavior or appearance, magical thinking). The highest risk of developing psychosis is from ages 15-30, but prodromal symptoms can appear earlier (10, 13).

## Neurocognition

During the course of schizophrenia, psychological testing or observation of the patient can reveal deficits in neurocognitive functions, varying in degrees of severity and range. Impairments in neurocognitive functions may be considered to be an indication of vulnerability for psychotic disorder (5). Neurodevelopmental and neurodegenerative models try to explain the pathomechanism of these changes in cognitive efficiency. Many studies have shown that deficits in cognitive functions are specific not only to full-blown schizophrenia, but also to UHR.

A large longitudinal study conducted in America (NAPLS) on a UHR group and patients with FP suggested that people with prodromal symptoms are already dealing with neurocognitive difficulties, but they are less severe than in people with a diagnosis of FP (20). People in the prodromal phase of schizophrenia generally show levels of impairments intermediate between schizophrenia patients and healthy controls (6).

A study conducted by a team from Switzerland found evidence of impairment in auditory working memory, verbal fluency, processing speed, and declarative verbal memory in a UHR group (21). A meta-analysis of 19 studies, comprising a total of 1,188 UHR subjects, found that prodromal symptoms of schizophrenia are associated with small to moderate neurocognitive deficits in general intelligence, executive functions, verbal fluency, attention, visual and verbal memory, and working memory (1).

Individuals who transitioned to full-blown psychosis had worse scores in verbal fluency, verbal and visual memory, and working memory (22). A general tendency in the severity of cognitive impairments in the schizophrenia spectrum is that if psychopathological symptoms last longer, the impairments are deeper.

It should be noted that impairments in general intelligence, attention, and visual and verbal memory are small to moderate in UHR, while they are moderate in FP, and deep in CHS (22, 23). Executive functions, verbal fluency, and working memory tend to get worse in the prodromal phase of psychosis and present in the form of small to moderate impairments, but then in FP and CHS these impairments last on a moderate level (23).

Processing speed index, measured in neurocognitive tests by symbol or digit coding speed, is the most differentiating factor between a healthy control group and schizophrenia spectrum patients (24, 25). These difficulties are considered to be the core neurocognitive impairments in schizophrenia (24). Deficits in processing speed appear at the very beginning of the psychotic process. Data have shown that there are significant differences in symbol coding speed between siblings at UHR of psychosis who later develop psychotic symptoms and those who do not (25).

The relationship between the onset of symptoms and the presence of different cognitive deficits can be two-sided. In the neurodegenerative model, psychotic symptoms cause typical brain dysfunctions. In this model, the severity and extent of cognitive impairments will increase with the duration and severity of clinical symptoms of UHR for psychosis. This also explains why we observe greater cognitive deficits at each stage of the schizophrenia continuum. But on the other hand, impairments in individual cognitive functions may have a significant impact on the development of psychotic and quasi-psychotic symptoms in UHR people. For example, impairments in attention and processing speed lead to an unrealistic perception of one's surroundings and strengthen the delusional attitude. Disturbances in cognitive functions can also have a negative impact on self-esteem and social relations. This can lead to social isolation and the development of negative symptoms.

The findings of Bora et al. (7) suggest that neurocognitive impairments are already established before the development of the UHR condition, which supports the neurodevelopmental model of schizophrenia. This theory implies that specific pathological processes disturb the developmental trajectory which, in the future, causes functional disorders and increased risk of developing a psychotic disorder. Results of the longitudinal population-based cohort study conducted by Mollon et al. (26) indicate that patients with psychotic disorder, unlike patients with psychosis with depression, psychotic experiences, or depression, present progressive deficits in IQ. Cognitive impairments in this group are already present in the first two decades of life. These findings suggest that cognitive deficits associated with psychosis may be the product of increasing lags across different critical developmental periods. Another longitudinal study confirming the neurodevelopmental model indicates that baseline cognitive impairment is a significant factor differentiating UHR individuals who present more severe symptoms from healthy controls and people who were diagnosed with UHR but whose symptoms are in remission (27).

In order to thoroughly investigate the cause of cognitive deficits in the UHR group, numerous neuroimaging studies have been conducted. A systematic review and meta-analysis performed in 2018 by a research group from China showed that data collected in different studies reveals a specific neurophysiology in UHR people (28). Structural abnormalities, such as in gray matter volume and cortical thickness of the thalamocortical circuit, are characteristic of UHR people and can be considered to be a marker of transition to psychosis (28). The very important role of prefrontal volume loss in the development of structural and functional brain changes has been demonstrated by a team from Melbourne University (29). Their research also found that UHR patients who developed a full psychotic episode had increased brain contraction in the right prefrontal region (29). Further discoveries regarding cognitive functioning and neurophysiological changes in individuals at ultra-high risk of psychosis would be beneficial, especially regarding the relation between neurocognitive functions and responses to treatment.

## Social Cognition

In trying to understand the relationship between social cognition and UHR, we shall first briefly look at the specifics of psychotic disorders with particular attention devoted to schizophrenia. Studies indicate that patients with a diagnosis of schizophrenia score worse on tasks assessing emotion perception, theory of mind (ToM), adaptive attributional styles, social perception, and social knowledge (30). In addition to social cognitive deficits, negative symptoms of psychosis such as lack of desire to form relationships, poverty of speech, and lack of motivation may impact social interactions. Various types of training in social skills have been created to help people with schizophrenia deal with their limitations (31, 32).

Researchers, based on their knowledge of relationships between social cognition and psychotic disorders, have conducted several studies focused on social cognition in UHR. A meta-analysis of 17 studies showed that general impairments in social cognition are typical in the UHR group (30). They discuss a study conducted by Addington and his team in which they proved that patients identified as UHR get significantly worse results on tasks involving emotion recognition (30). A study performed by a team from Korea confirmed these results and found that people in an at-risk state processed facial configuration at a lower level than the healthy control group, as do schizophrenia patients (33). This effect did not extend to processing facial features (33). Overall, research confirms mild impairments in emotion recognition in UHR people. The abilities to recognize emotions based on faces and voice are similarly impaired in FP, but these skills are significantly worse in CHS. There is evidence that, like schizophrenia patients, people in a UHR state have greater difficulty recognizing negative facial affect, for example emotions such as fear, disgust, or anger (34).

The inspiration to look for a relationship between social cognition deficits and UHR was reports suggesting that patients with autism spectrum disorders (ASD) are at greater risk of developing affective and nonaffective psychoses (35). ASD and different disorders from the schizophrenia spectrum are related to similar levels of social cognition impairments (36, 37). Despite the fact that the co-diagnosis of ASD and schizophrenia is controversial and is still a topic of debate, researchers are studying the relationship between ASD symptoms and psychotic symptoms because of some commonalities between these disorders. Research on the role of ASD symptoms in the context of psychotic symptoms has also been conducted with UHR groups (37, 38). Although the results of the study conducted by Foss-Feig et al. suggest that there are no differences between patients with UHR with and without ASD in baseline psychosis symptoms and conversion rates, further research on this topic could furnish significant insights because of the similar social cognition deficits in both groups (38).

Another skill which is very important in social functioning is mentalizing, described in Theory of Mind (ToM) as the ability to be aware of the mental states of other people, such as moods or emotions, and their impact on behavior. There is evidence that impairments in metalizing are present at every stage of psychotic spectrum disorders, but it is still unknown whether the severity of these impairments increases at each stage (39). However, Debbané et al. suggest that ideas of reference and odd beliefs, understood as disturbances in the process of mentalization, are more likely to be present in early stages of psychosis (39). On the other hand, a lack of changes to the ability to metalize is recognized as a protective factor against vulnerability for psychosis (39).

It has also been shown that there is a correlation between atypical brain activation patterns during tasks involving monitoring the reality and perception of self and others and manifestation of sociotype in adolescence (39). Regarding ToM, a meta-analysis of 17 studies showed that there are significant moderate deficits in verbal ToM in UHR samples (9). A study conducted in Denmark showed that people in the UHR phase scored significantly worse than healthy controls on a ToM measure and a global measure of emotion recognition (34). Ayesa-Arriola et al. (40) showed that deficits in ToM are a trait of schizophrenia. These difficulties are present from the onset of psychosis, remain throughout the illness, and are not associated with clinical symptoms (40). There is some evidence that there are differences in measures of attributional bias between UHR groups and healthy controls, but other studies did not find such differences (9, 30, 34). Specific attributional biases are characteristic of schizophrenia patients, but further research is needed to identify whether there is a correlation between frequency and types of attributional bias on each psychotic disorder spectrum.

An interesting issue is the impact of UHR symptoms on social cognition. A study conducted by a team from the Netherlands found data suggesting that adolescents in a UHR state have problems identifying and verbalizing their own emotions and, furthermore, the level of difficulty was related to the severity of high risk symptoms (41). A study by Shim et al. indicated that the duration of prodromal symptoms was correlated with impaired social skills and general symptoms were significantly related to lower levels of “independence-competence” (42). Intensification of positive and negative symptoms did not affect social competences (42). Another study showed that negative symptoms are a significant predictor of impairments in social skills (34). Moreover, negative symptoms, especially experiential ones such as avolition and anhedonia, tend to have a strong impact on functioning in UHR individuals (34). There is evidence that anhedonia may be a predictor of transition to full-blown psychosis (34). The severity and type of social cognition impairments are not themselves correlated with frequency and time of transition from UHR to psychosis during adolescence (39). However, data about relationships between social cognition and different stages of psychotic disorders are still limited and further research needs to be conducted to understand this topic more thoroughly.

## Possibilities for Treatment

Diagnostic criteria, evidence from research, brain mechanism models, case studies and other methods of gathering knowledge about specific psychiatric conditions are investigated, among other reasons, to find effective methods of treating people who seek help. As the prodromal phase of psychosis is not recognized as a disorder in ICD or DSM criteria, therapeutic interactions should be focused on prevention of conversion to psychosis or other disorders and should include actions which aim to improve quality of functioning.

Taking into consideration the guidance for early detection, the European Psychiatric Association (EPA) has developed evidence-based recommendations for interventions for individuals at UHR for psychosis (43). Much research indicates that both psychological and pharmacological interventions can be beneficial in the UHR group (43). The EPA recommends cognitive-behavioral therapy (CBT) as the first-choice intervention in the prodromal phase of psychosis; this can be complemented by pharmacological interventions with low dose second-generation antipsychotic drugs (43). The decision to add pharmacology to a treatment should be considered when the severity of symptoms limits the efficacy of CBT (43). Antipsychotic medications should only be given in exceptional circumstances because of, inter alia, their side-effects. Proper selection and implementation of therapy can postpone or even prevent the development of full-blown disorders.

Many studies indicate that CBT is the most effective method of providing help to people at risk of psychosis. A study conducted in 2012 in the Netherlands indicated that CBT can reduce the transition to psychosis by about 50% (44). Another study showed that after 4 years of follow-up, CBT is still effective at preventing transition from UHR to FP (45). Moritz et al. (15) highlight the controversy regarding the effectiveness of interventions in the UHR state. Despite the fact that there are studies showing the effectiveness of CBT and CBT combined with pharmacotherapy in UHR state, there are also other data suggesting that these forms of intervention cannot prevent transition to psychosis (15). There are also data suggesting that there are no advantages of specialized treatment over need-based treatment in the UHR state (15). Specialized treatment for people in the UHR state, especially when it is so named, is itself controversial because of the risk of stigmatization. Stigma—fear of becoming psychotic—may lead to depression and increase a risk of suicide (15). Interventions for people at UHR should rather focus on current problems and provide knowledge about mental health in general, not only about psychotic disorders.

The risk of transition to psychosis is highest in the first year after diagnosis of UHR (45). Therapeutic intervention should be offered in the first half year of prodromal symptoms, as it increases the effectiveness of therapy (45). Cognitive-behavioral therapy may be effective in the treatment of the UHR because, inter alia, during the therapy a person learns how to monitor emotions, test their own thoughts, control behavior, and see the interactions between these three elements. Therapy offers the opportunity to share, find understanding, and become able to challenge cognitive biases in a safe environment. In UHR for psychosis, we can observe some specific elements in cognition: e.g., the presence of dysfunctional metacognitive beliefs that can lead to misjudging reality and others' intentions as well as cause difficulty in dealing with stressful life events (11). Young people in the prodromal phase of psychosis tend to worry about the condition of their memory and attention and view their thoughts as uncontrollable and dangerous (46). In the treatment of patients who deal with dysfunctional beliefs, metacognitive training—a form of CBT—or other cognitive techniques may be beneficial (11).

Despite the fact that the EPA recommendations indicate the use of CBT or CBT combined with pharmacotherapy, other treatment opportunities have been offered and tested for effectiveness in the UHR group. One such promising method is cognitive remediation (CR). CR is an intervention based on behavioral training composed of various tasks focused on improving cognitive functioning (47, 48). It is an evidenced-based intervention in schizophrenia which can improve cognitive functioning and, with it, the quality of everyday functioning (47). The previously mentioned neurodegenerative and neurodevelopmental model of psychosis suggests that such interventions could also be effective in the UHR group. There are studies indicating that CR may improve different domains of cognitive functions, such as verbal memory, visual memory, attention, processing speed, facial emotion recognition, executive functioning, and social functioning in people at UHR. However, it has not been confirmed that this form of intervention influences global measures of cognition, clinical symptoms, or functioning (49, 50). More research on the effectiveness of CR in UHR is needed, but it may be a promising intervention option.

## Discussion

In modern psychiatry, more and more time is being devoted to developing a detailed understanding of the different stages of the spectrum of mental disorders. There have been many reports on the ultra high risk (UHR) for psychosis state in recent years, giving us a better understanding of the cognitive and social functioning of people in this group. Knowledge about the difficulties faced by people at UHR enables us to tailor therapies appropriately. Due to the specificity and variability of prodromal symptoms and the sensitivity of patients, this issue should be treated with exceptional dedication and caution at the same time.

Impairments in cognitive functions are described in the UHR state and some studies suggest their presence even before the onset of prodromal symptoms. From a clinical perspective, it seems appropriate to use cognitive training or remediation in patients with prodromal symptoms. This form of intervention can not only improve patients' quality of life, but also slow or prevent the development of symptoms. Based on the neurodegenerative model of psychotic disorders, future research should focus on the relationship between therapeutic interactions and the severity of cognitive deficits. When assessing cognitive functions in patients from the UHR group, it is worthwhile to conduct follow-up research.

Some studies have suggested that social cognition impairments are characteristic of the UHR state, but data on specific skills are still limited. Research in this area should be continued. It seems particularly interesting to identify the specific relationships between difficulties in social cognition and global functioning. Knowledge about impairments in social cognition in the UHR state implies that therapeutic interactions should also address strengthening social competences.

The UHR state is not included in any leading classification of psychiatric disorders. Nevertheless, the diagnostic criteria for UHR were chosen to identify people who are more likely to develop a psychotic disorder. On the one hand, early detection of UHR enables the monitoring of the patient's condition and the implementation of appropriate interventions to stop or slow down the development of symptoms. On the other hand, all clinicians should keep in mind the risk of stigmatization of UHR patients, which may have fatal consequences. The effects of stigmatization may include a decrease in self-esteem, social isolation, hopelessness, increased use of avoidance strategies, as well as depression and anxiety symptoms (13, 51). Path analysis indicates that self-labeling and stress related to stigma is a valuable predictor of suicide (51). Clinicians must both treat mental illness and help maintain good mental health; therefore, when planning interventions in the UHR group, both the positive and negative consequences of diagnosis should be taken into account. It should be considered whether the use of the term UHR for psychosis should be applied directly to the patient at all. Perhaps a better approach would be to address the patient's current problems and symptoms without predicting the development of severe mental illness. Even though one study of 55 patients and 50 professionals found that patients were less likely than professionals to believe that the terms UHR and APS were at risk of stigmatization, due to, inter alia, the small size of the study group; further research on the topic is needed to draw firm conclusions on this point (52). It is also important to note that patients with a family history of psychosis were more likely to associate the term UHR with stigma and agreed that this term should be changed (52). Certainly the stigmatization effect in UHR should be taken into account in the process of diagnosis and therapy to avoid iatrogenic effects.

Evidence-based therapeutic methods, such as CBT or CBT combined with pharmacotherapy, are recommended by the EPA as the interventions for the UHR state. Research results suggest that this intervention might be effective in preventing transition to full-blown psychosis. However, it is worth mentioning that there is no clear agreement on these recommendations. Research in this area should definitely be continued. Clinical experience and a holistic view of people suggest a direction of research that focuses not on creating therapies specifically for UHR patients, but rather on adjusting interventions to the current needs of the patient. Therapy should focus on reducing subjective discomfort, teaching adaptive techniques for managing stress, improving social skills, and undermining cognitive biases. Even if it might sometimes be impossible to fully prevent the development of an illness, any delay could be very beneficial from the perspective of brain mechanisms, social functioning, and relations with others. Furthermore, early interventions include psychoeducation and provide the abilities necessary to deal with the disease, so they can ensure a milder course of the disorder.

The efficacy of different methods and therapeutic techniques still needs to be tested in UHR patients. Current evidence is promising, but it is still worthwhile to investigate the effectiveness of other methods. Young people in an at-risk state can probably respond well to group therapy or modern forms of treatment using techniques such as computer training, virtual reality, or mobile applications. Despite the recommendations and evidence from research, early interventions are not as widely used as they should be. A study conducted in England showed that only 53% of 50 medical teams included these treatments in their service and provided them for at least 12 months (53).

Despite knowledge about the UHR condition and the effectiveness of therapy, early interventions are still not as widely used as they should be. There is a pressing need to raise awareness about UHR symptoms and possible methods of treatment.

## Conclusion

To conclude, UHR for psychosis is the condition of being at risk of developing a psychotic disorder in which attenuated psychotic symptoms, frank, transient psychotic symptoms, or genetic risk and deterioration syndrome are present. It most often affects young people, and even when it does not lead to a transition to full-blown psychosis or some other psychiatric disorder, it impairs neurocognitive functions and social cognitive functioning. Deficits in cognitive functions such as general intelligence, memory, processing speed, attention, executive functions, and verbal fluency have been demonstrated by different studies. These impairments tend to be established before the appearance of prodromal symptoms, which supports the neurodevelopmental model of psychotic disorders. Structural and functional changes in brain activity in the prodromal phase of psychosis, such as gray matter volume, cortical thickness of the thalamocortical circuit, and prefrontal volume loss, have been shown by neuroimaging studies. UHR is also associated with difficulties in emotion perception based on face and voice recognition, mentalizing, and theory of mind. Early diagnosis, symptom monitoring, and early therapeutic interventions are key in people experiencing UHR. Avoiding stigmatization is extremely important during the diagnosis and treatment of people from the UHR group.

## Author Contributions

KR-O and ET was involved in the paper design, managed literature searches and analyses, and wrote the first draft of the manuscript. JS and MM was a supervisor and corrected the manuscript. KW corrected the manuscript. All authors contributed to and have approved the final manuscript.

## Funding

This paper was funded by the Polish Minister of Science and Higher Education's program named Regional Initiative of Excellence in 2019–2022, grant number 002/RID/2018/2019 to the amount of 12 000 000 PLN.

## Conflict of Interest

The authors declare that the research was conducted in the absence of any commercial or financial relationships that could be construed as a potential conflict of interest. The handling editor GD declared a past co-authorship with one of the authors.

## Publisher's Note

All claims expressed in this article are solely those of the authors and do not necessarily represent those of their affiliated organizations, or those of the publisher, the editors and the reviewers. Any product that may be evaluated in this article, or claim that may be made by its manufacturer, is not guaranteed or endorsed by the publisher.

## References

[1] Fusar-Poli P Bonoldi I Yung AR Borgwardt S Kempton MJ Valmaggia L. Predicting psychosis: meta-analysis of transition outcomes in individuals at high clinical risk. Arch Gen Psychiatry. (2012) 69:220–9. 10.1001/archgenpsychiatry.2011.1472 22393215

[2] Simon AE Velthorst E Nieman DH Linszen D Umbricht D de Haan L. Ultra high-risk state for psychosis and non-transition: a systematic review. Schizophr Res. (2011) 132:8–17. 10.1016/j.schres.2011.07.002 21784618

[3] Fusar-Poli P Rocchetti M Sardella A Avila A Brandizzi M Caverzas E. Disorder, not just state of risk: meta-analysis of functioning and quality of life in people at high risk of psychosis. Br J Psychiatry. (2015) 207:198–206. 10.1192/bjp.bp.114.157115 26329563

[4] Kraepelin E. Dementia Praecox and Paraphrenia. Chicago, IL: Chicago Medical Journal Co. (1919).

[5] Bleuler E. Dementia Praecox or the Group of Schizophrenias. New York, NY: International Universities Press (1950).

[6] Bang M Kim KR Song YY Beak S Lee E Ann SK. Neurocognitive impairments in individuals at ultra-high risk for psychosis: who will really convert? Aust N Z J Psychiatry. (2015) 49:462–70. 10.1177/0004867414561527 25425742

[7] Bora E Lin A Wood SJ Yung AR McGorry PD Pantelis C. Cognitive deficits in youth with familial and clinical high risk to psychosis: a systematic review and meta-analysis. Acta Psychiatr Scand. (2014) 130:1–15. 10.1111/acps.12261 24611632

[8] Bora E Murray RM. Meta-analysis of cognitive deficits in ultra- high risk to psychosis and first- episode psychosis: do the cognitive deficits progress over, or after, the onset of psychosis? Schizophr Bull. (2014) 40:744–55. 10.1093/schbul/sbt085 23770934 PMC4059428

[9] Van Donkersgoed RJ Wunderink L Nieboer R Aleman A Pijnenborg GH. Social cognition in individuals at ultra-high risk for psychosis: a meta-analysis. PLoS One. (2015) 10:e0141075. 10.1371/journal.pone.0141075 26510175 PMC4624797

[10] Fusar-Poli P Borgwardt S Bechdolf A Addington J Riecher-Rössler A Schultze-Lutter F. The psychosis high-risk state: a comprehensive state-of-the-art review. JAMA Psychiatry. (2013) 70:107–20. 10.1001/jamapsychiatry.2013.269 23165428 PMC4356506

[11] Yung AR. Treatment of people at ultra-high risk for psychosis. World Psychiatry. (2017) 16:207–8. 10.1002/wps.20424 28498602 PMC5428169

[12] McGlashan T Walsh B Woods S. Structured Interview for Psychotic Symptoms. PRIME Research Clinic. New Haven, CT: Yale School of Medicine (2001).

[13] Nelson B Yuen K Yung AR. Ultra high risk (UHR) for psychosis criteria: Are there different levels of risk for transition to psychosis? Schizophr Res. (2011) 125:62–8. 10.1016/j.schres.2010.10.017 21074975

[14] Kline E Wilson C Ereshefsky S Tsuji T Schiffman J Pitts S. Convergent and discriminant validity of attenuated psychosis screening tools. Schizophr Res. (2012) 134:49–53. 10.1016/j.schres.2011.10.001 22036199

[15] Mortiz S Gaweda Ł Heinz A Gallinat J. Four reasons why early detection centers for psychosis should be renamed and their treatment targets reconsidered: we should not catastrophize a future we can neither reliably predict nor change. Psychol Med. (2019) 49:2134–40. 10.1017/S0033291719001740 31337458

[16] McGorry PD Hartmann JA Spooner R Nelson B. Beyond the “at risk mental state” concept: transitioning to transdiagnostic psychiatry. World Psychiatry. (2018) 17:133–42. 10.1002/wps.20514 29856558 PMC5980504

[17] Yung AR Phillips LJ McGorry PD McFarlane CA Francey S Harrigan S. Prediction of psychosis: a step towards indicated prevention of schizophrenia. Br J Psychiatry. (1998) 172:14–20. 10.1192/S0007125000297602 9764121

[18] World Health Organization. ICD-10: Schizophrenia. (2021). Available online at: https://icd.who.int/browse10/2019/en#/F23

[19] Schimmelmann BG Michel C Martz-Irngartinger A Linder C Schultze-Lutter F. Age matters in the prevalence and clinical significance of ultra-high-risk for psychosis symptoms and criteria in the general population: findings from the BEAR and BEARS-kid studies. World Psychiatry. (2015) 14:189–97. 10.1002/wps.20216 26043337 PMC4471976

[20] Seidman LJ Giuliano AJ Meyer EC Addington J Cadenhead KS Cannon TD. Neuropsychology of the prodrome to psychosis in the NAPLS consortium: relationship to family history and conversion to psychosis. Arch Gen Psychiatry. (2010) 67:578–88. 10.1001/archgenpsychiatry.2010.66 20530007 PMC3332118

[21] Simon AE Cattapan-Ludewig K Zmilacher S Arbach D Gruber K Dvorsky DN. Cognitive functioning in the schizophrenia prodrome. Schizophrenia Bull. (2007) 33:761–71. 10.1093/schbul/sbm018 17412711 PMC2526133

[22] Fusar-Poli P Deste G Smieskova R Barlati S Yung AR Howes O. Cognitive functioning in prodromal psychosis: a meta-analysis. Arch Gen Psychiatry. (2012) 69:562–71. 10.1001/archgenpsychiatry.2011.1592 22664547

[23] Sponheim SR Jung RE Seidman LJ Mesholam-Gately RI Manoach DS O'Leary DS. Cognitive deficits in recent-onset and chronic schizophrenia. J Psychiatr Res. (2010) 44:421–28. 10.1016/j.jpsychires.2009.09.010 19878956 PMC3940967

[24] Kelleher I Murtagh A Clarke MC Murphy J Rawdon C Cannon M. Neurocognitive performance of a community-based sample of young people at putative ultra high risk for psychosis: support for the processing speed hypothesis. Cogn Neuropsychiatry. (2013) 18:9–25. 10.1080/13546805.2012.682363 22991935

[25] Leeson VC Barnes TR Harrison M Matheson E Harrison I Mutsatsa SH. The relationship between IQ, memory, executive function, and processing speed in recent-onset psychosis: 1-year stability and clinical outcome. Schizophr Bull. (2010) 36:400–9. 10.1093/schbul/sbn100 18682375 PMC2833117

[26] Mollon J David AS Zammit S Lewis G Reichenberg A. Course of cognitive development from infancy to early adulthood in the psychosis spectrum. JAMA Psychiatry. (2018) 75:270–9. 10.1001/jamapsychiatry.2017.4327 29387877 PMC5885954

[27] Lam M Lee J Rapisarda A See YM Yang Z Lee SA. Longitudinal cognitive changes in young individuals at ultrahigh risk for psychosis. JAMA Psychiatry. (2018) 75:929–39. 10.1001/jamapsychiatry.2018.1668 30046827 PMC6142925

[28] Ding Y Ou Y Pan P Shan X Chen J Liu F. Brain structural abnormalities as potential markers for detecting individuals with ultra-high risk for psychosis: a systematic review and meta-analysis. Schizophr Res. (2019) 209:22–31. 10.1016/j.schres.2019.05.015 31104914

[29] Sun D Phillips L Velakoulis D Yung A McGorry PD Wood SJ. Progressive brain structural changes mapped as psychosis develops in ‘at risk'individuals. Schizophr Res. (2009) 108:85–92. 10.1016/j.schres.2008.11.026 19138834 PMC2670732

[30] Thompson AD Bartholomeusz C Yung AR. Social cognition deficits and the “ultra high risk” for psychosis population: a review of literature. Early Interv Psychiatry. (2011) 5:192–202. 10.1111/j.1751-7893.2011.00275.x 21726422

[31] Kurtz MM Mueser KT. A meta-analysis of controlled research on social skills training for schizophrenia. J Consult Clin Psychol. (2008) 76:491–504. 10.1037/0022-006X.76.3.491 18540742

[32] Granholm E Harvey PD. Social skills training for negative symptoms of schizophrenia. Schizophr Bull. (2018) 44:472–4. 10.1093/schbul/sbx184 29315427 PMC5890477

[33] Kim HS Shin NY Choi JS Jung MH Jang JH Kang DH. Processing of facial configuration in individuals at ultra-high risk for schizophrenia. Schizophr Res. (2010) 118:81–7. 10.1016/j.schres.2010.01.003 20133107

[34] Glenthøj LB Fagerlund B Hjorthøj C Jepsen JR Bak N Kristensen TD. Social cognition in patients at ultra-high risk for psychosis: what is the relation to social skills and functioning? Schizophr Res Cogn. (2016) 5:21–7. 10.1016/j.scog.2016.06.004 28740813 PMC5514303

[35] Selten JP Lundberg M Rai D Magnusson C. Risks for nonaffective psychotic disorder and bipolar disorder in young people with autism spectrum disorder: a population-based study. JAMA Psychiatry. (2015) 72:483–9. 10.1001/jamapsychiatry.2014.3059 25806797

[36] Barlati S Minelli A Ceraso A Nibbio G Silva RC Deste G. Social cognition in a research domain criteria perspective: a bridge between schizophrenia and autism spectra disorders. Front Psychiatry. (2020) 11:e806. 10.3389/fpsyt.2020.00806 33005149 PMC7485015

[37] Maat A Therman S Swaab H Ziermans T. The attenuated psychosis syndrome and facial affect processing in adolescents with and without autism. Front Psychiatry. (2020) 11:e759. 10.3389/fpsyt.2020.00759 32848934 PMC7416636

[38] Foss-Feig JH Velthorst E Smith L Reichenberg A Addington J Cadenhead KS. Clinical profiles and conversion rates among young individuals with autism spectrum disorder who present to clinical high risk for psychosis services. J Am Acad Child Adolesc Psychiatry. (2019) 58:582–8. 10.1016/j.jaac.2018.09.446 30797038 PMC7781438

[39] Debbané M Salaminios G Luyten P Badoud D Armando M Solida Tozzi A. Attachment, neurobiology, and mentalizing along the psychosis continuum. Front Hum Neurosci. (2016) 10:e406. 10.3389/fnhum.2016.00406 27597820 PMC4992687

[40] Ayesa-Arriola R Setién-Suero E Neergaard KD Ferro A Fatjó-Vilas M Ríos-Lago M. Evidence for trait related theory of mind impairment in first episode psychosis patients and its relationship with processing speed: a 3 year follow-up study. Front Psychol. (2016) 7:e592. 10.3389/fpsyg.2016.00592 27199826 PMC4854883

[41] Van Rijn S Schothorst P van't Wout M Sprong M Ziermans T van Engeland H. Affective dysfunctions in adolescents at risk for psychosis: emotion awareness and social functioning. Psychiatry Res. (2011) 187:100–5. 10.1016/j.psychres.2010.10.007 21094533

[42] Shim G Kang DH Sun Chung Y Young Yoo S Young Shin N Soo Kwon J. Social functioning deficits in young people at risk for schizophrenia. Aust N Z J Psychiatry. (2008) 42:678–85. 10.1080/00048670802203459 18622775

[43] Schultze-Lutter F Michel C Schmidt SJ Schimmelmann BG Maric NP Salokangas RK. EPA guidance on the early detection of clinical high risk states of psychoses. Eur Psychiatry. (2015) 30:405–16. 10.1016/j.eurpsy.2015.01.010 25735810

[44] Van der Gaag M Nieman DH Rietdijk J Dragt S Ising HK Klaassen RM. Cognitive behavioral therapy for subjects at ultrahigh risk for developing psychosis: a randomized controlled clinical trial. Schizophrenia Bull. (2012) 38:1180–8. 10.1093/schbul/sbs105 22941746 PMC3494039

[45] Ising HK Kraan TC Rietdijk J Dragt S Klaassen RM Boonstra N. Four-year follow-up of cognitive behavioral therapy in persons at ultra-high risk for developing psychosis: the Dutch early detection intervention evaluation (EDIE-NL) trial. Schizophr Bull. (2016) 42:1243–52. 10.1093/schbul/sbw018 26994397 PMC4988735

[46] Cotter J Yung AR Carney R Drake RJ. Metacognitive beliefs in the at-risk mental state: a systematic review and meta-analysis. Behav Res Ther. (2017) 90:25–31. 10.1016/j.brat.2016.12.004 27960094

[47] Vita A Barlati S Ceraso A Nibbio G Ariu C Deste G. Effectiveness, core elements, and moderators of response of cognitive remediation for schizophrenia: a systematic review and meta-analysis of randomized clinical trials. JAMA Psychiatry. (2021) 78:848–58. 10.1001/jamapsychiatry.2021.0620 33877289 PMC8058696

[48] Mak M Tyburski E Starkowska A Karabanowicz E Samochowiec A Samochowiec J. The efficacy of computer-based cognitive training for executive dysfunction in schizophrenia. Psychiatry Res. (2019) 279:62–70. 10.1016/j.psychres.2019.06.041 31302353

[49] Glenthøj LB Hjorthøj C Kristensen TD Davidson CA Nordentoft M. The effect of cognitive remediation in individuals at ultra-high risk for psychosis: a systematic review. NPJ Schizophr. (2017) 3:e20. 10.1038/s41537-017-0021-9 28560266 PMC5441569

[50] Glenthøj LB Mariegaard LS Fagerlund B Jepsen JR Kristensen TD Wenneberg C. Cognitive remediation plus standard treatment versus standard treatment alone for individuals at ultra-high risk of developing psychosis: results of the FOCUS randomised clinical trial. Schizophr Res. (2020) 224:151–8. 10.1016/j.schres.2020.08.016 32873460

[51] Xu Z Müller M Heekeren K Theodoridou A Metzler S Dvorsky D. Pathways between stigma and suicidal ideation among people at risk of psychosis. Schizophr Res. (2016) 172:184–8. 10.1016/j.schres.2016.01.048 26843510

[52] Kim SW Polari A Melville F Moller B Kim JM Amminger P. Are current labeling terms suitable for people who are at risk of psychosis? Schizophr Res. (2017) 188:172–7. 10.1016/j.schres.2017.01.027 28117104

[53] Stain HJ Mawn L Common S Pilton M Thompson A. Research and practice for ultra-high risk for psychosis: a national survey of early intervention in psychosis services in England. Early Interv Psychiatry. (2019) 13:47–52. 10.1111/eip.12443 28612979

